# Non-medical prescription stimulant use to improve academic performance among Australian university students: prevalence and correlates of use

**DOI:** 10.1186/s12889-018-6212-0

**Published:** 2018-11-19

**Authors:** Jayne Lucke, Charmaine Jensen, Matthew Dunn, Gary Chan, Cynthia Forlini, Sharlene Kaye, Bradley Partridge, Michael Farrell, Eric Racine, Wayne Hall

**Affiliations:** 1 Australian Research Centre in Sex, Health and Society, School of Psychology and Public Health, College of Science, Health and Engineering, Building NR6, La Trobe University, Bundoora, VIC 3086 Australia; 2 School of Public Health, The University of Queensland, Herston, Brisbane, QLD Australia; 3 The University of Queensland Centre for Youth Substance Abuse Research, St Lucia, Brisbane, QLD Australia; 4 School of Health and Social Development, Deakin University, Geelong, Australia; 5 National Drug and Alcohol Research Centre, University of New South Wales, Sydney, NSW Australia; 6 Sydney Health Ethics, University of Sydney, Sydney, NSW Australia; 7 Research Development Unit, Caboolture Hospital, Caboolture, QLD Australia; 8 School of Clinical Medicine, Prince Charles Hospital Northside Clinical Unit, The University of Queensland, Brisbane, QLD Australia; 9 Pragmatic Health Ethics Research Unit, Institut de recherches cliniques de Montréal, Montréal, Quebec, Canada

**Keywords:** Prescription stimulants, Cognitive enhancement, academic performance, Caffeine, University students, Australia, Prevalence, Correlates

## Abstract

**Background:**

Some university students consume pharmaceutical stimulants without a medical prescription with the goal of improving their academic performance. The prevalence of this practice has been well documented in the US, but less so in other countries. The potential harms of using prescription stimulants require a better understanding of the prevalence of this practice within Australian universities.

**Methods:**

An internet survey of 1136 Australian students was conducted in 2015 in three large Australian universities. Students were asked about their personal use of prescription stimulants, attitudes and experiences with prescription stimulants. They were also asked about their use of caffeine, energy drinks and illicit drugs to enhance their academic performance.

**Results:**

Lifetime self-reported use of stimulant medication to improve academic performance was 6.5, and 4.4% in the past year. Students were far more likely to report using coffee and energy drinks (41.4 and 23.6% respectively, lifetime use) than prescription stimulants to help them study and complete university assessments. Non-medical use of prescription stimulants was strongly associated with a history of illicit drug use.

**Conclusion:**

The prevalence of nonmedical prescription stimulant use to improve academic performance is low among university students in Australia, especially when compared with their use of coffee and energy drinks.

## Background

The increasing prescription of stimulant medications to treat Attention-Deficit Hyperactivity Disorder (ADHD) has led to concerns about the diversion and misuse of these medications by young people without a diagnosis of ADHD [1]. In particular, there has been considerable attention directed towards the non-medical use of prescription stimulants such as methylphenidate (Concerta, Ritalin), dextroamphetamine and amphetamine (Adderall) and modafinil (Modavigil) among North American college students as “study aids” to improve academic performance. Although relatively little is known about the prevalence of non-medical prescription stimulant use by Australian university students, there is some evidence that Australian university students use prescription stimulants non-medically as a way of coping with the demands of study [2, 3]. Qualitative studies with Australian university students show that many are aware that some students use prescription stimulants as “study drugs” [4, 5]. The harms related to non-medical prescription stimulant use can be considerable [1], and it is therefore necessary to have an understanding of this important public health issue [6].

Recent findings from the US National Surveys on Drug Use and Health indicate that 6.6% of adults used prescription stimulants on average over a year, and of the small proportion of those who misuse stimulants (around 2%) most do so to help them remain alert or concentrate [7]. While the problem of prescription stimulant misuse may not be significant in the general population, research suggests a high prevalence of diversion and misuse of prescription stimulants prescribed for ADHD in adolescent and young student populations [1, 8]. There has been a particular focus on prescription stimulant misuse among students because of evidence that prescription stimulant misuse is higher in college students than among young people who do not attend college [9].

Much of the research on nonmedical prescription stimulant use among university students has come from the US, with studies including two recent reviews indicating a lifetime prevalence ranging from 5 to 43% [10–12]. The wide variations in estimates of prevalence were attributable to different inclusion criteria and to methodological differences between the studies. However, national surveys of US college students have reported a prevalence of non-medical prescription stimulant use between 3 and 6% [13–16]. A survey of 877 UK and Irish students in 2012 found that only 0.3–4% were regular past or current users of modafinil, methylphenidate or Adderall for cognitive enhancement and 10% reported lifetime use [17]. These findings are similar to an Australian survey of 1729 university students which reported a lifetime prevalence 10.9% [3]. Other international studies have reported a higher prevalence of use, such as in Iceland (13%) [18] and Puerto Rico (23.3%) [19]. The range of prevalence reported internationally highlights the need to collect good Australian data as it is difficult to generalise from the results of studies in other countries.

There is a high prevalence of prescription stimulant use among those who drink alcohol [20] and those who frequently consume drugs [21]. Among young people dexamphetamine is seen as ‘safe’ because it is a pharmaceutical [22]. However, studies show that the most commonly reported motivation for prescription stimulant use among students is to help them concentrate and focus, and stay awake to complete assignments or study for exams [3, 18, 23–25]. Given reported prescription stimulant use among student groups, further information about the motivations for such use is needed to inform Australian policy and practice. The aim of this study was to investigate non-medical prescription stimulant use by Australian university students to improve academic performance, and specifically to report on the prevalence and correlates of prescription stimulant use for this purpose.

## Methods

Students from three universities in Australia aged between 18 and 29 years were invited through targeted emails and university-based advertising to participate in an online survey in 2015 exploring substance use for academic improvement. Due to the requirements of the three universities it was not possible to send reminders to students who did not respond to the initial advertising or email invitation. The survey defined prescription stimulants to improve academic performance as “The *non-medical* use of prescription stimulants (such as Ritalin [methylphenidate], Adderall [dextroamphetamine] and modafinil [Provigil] without a prescription from a doctor) by students in an attempt to enhance their alertness, concentration, motivation or overall productivity.” Participants were reminded where relevant that use of these drugs for recreational, medical or other non-study purposes did not count as use for improving academic performance. A direct hyperlink to the survey was posted on student association websites, degree or subject portals, or emailed.

The online survey was created with Checkbox Inc. Version 6.7. All data generated was stored securely with password protection. Each institution was provided with a unique URL link to their survey. Participants received an information sheet and provided written consent online before they could undertake the survey. Students who read the survey information sheet without completing the survey were considered an incomplete response. A survey response was considered complete only when the respondent clicked the “finish” button at the end of the survey. Participants could save and exit the survey at any time by closing their browser. Incentives were provided to increase student participation. Students could opt to participate in a prize-pool at the end of the survey consisting of 1 × Apple iPad Air 16GB, 2×Apple iPad Minis 16GB, 3 × $50 Coles Myer vouchers, and 4×$20 Coles Myer vouchers. Prizes were drawn at the end of the academic semester. A random number generator was used to draw prize winners who met the inclusion criteria.

The online survey was constructed by the authors on the basis of previous research in this field, with particular attempts to address the limitations of previous surveys. The survey consisted of seven sections: i) demographics (e.g., age, sex, employment status); ii) education (e.g., years studying, study load, current Grade Point Average; discipline area); iii) suspected and diagnosed mental and physical health concerns (e.g., “Do you have any psychological health concerns? For example, anxiety, depression or attention deficit hyperactivity disorder”); iv) substance use for recreational purposes; v) attitudes toward the use of prescription stimulants to improve academic performance (e.g., “Students using prescription stimulants to help them study is acceptable” and “Prescription stimulants improve your study or academic performance”); vi) experiences with the use of prescription stimulants to improve academic performance (e.g., “Have you ever used substances to help you perform better at university? These substances may be anything from coffee or tobacco to prescription stimulants or street drugs used with the intention to help you study, work on assignments or take examinations”); and vii) substances used to improve academic performance consisting of over-the-counter products (e.g., coffee, energy drinks), prescription medication (modafinil, benzodiazepines), or illicit/street drugs (e.g., cocaine, ice).

IBM SPSS Statistics 23 software was used to generate descriptive statistics, frequencies, and means. The data was weighted by sex in order to represent the sex ratios for each participating university in accordance with each institution’s 2015 student population numbers. Binary logistic regression was used to explore correlates of prescription stimulant use to improve academic performance that included age, sex, stimulant prescription, recreational illicit drug use, frequency of alcohol use and association with students who used prescription stimulants to improve academic performance. All variables were entered as one block in the model.

Ethical approval was obtained from the University of Queensland Behavioural and Social Sciences Ethical Review Committee (Approval Number: 2014001403). Permission was also granted for data collection at each participating university.

## Results

### Characteristics of the sample

A total of 2063 students viewed the survey. Of these 850 students viewed the information sheet but did not provide consent, 41 students provided consent but did not complete any survey items, and 34 responses were removed because the respondents were outside the specified age range. Two responses were also removed as they did not provide their sex and were not included in analyses weighted by sex. Therefore, the final sample comprised 1136 university students. The proportion of participants from each of the three participating universities were 24.4, 37.6, and 38.0%. Compared to the university population in general the sample is slightly over representative of female students (62.2% in this survey compared with 57.0% in the general student population) but had a similar proportion of international students (14.4%) [26]. Sample characteristics and their association with prescription stimulant use are presented in Table 1. Table 1 also shows the results of weighted analyses performed to examine the association between life time prescription stimulant use and sample characteristics. *P*-values were calculated based on designed based F-statistics.

**Table 1:** Sample characteristics and their association with prescription stimulant use (*N* = 1136)

		Life time Prescription stimulant use (Weighted mean/ prevalence)		
	Overall sample (Unweighted descriptive)	No	Yes	p*
Characteristics	M (SD)	M	M	
Age	21.29 (2.78)	21.26	22.14	.005
	*n* (%)	%	%	
Sex				
Female	706 (62.2)	41.3	69.4	< .001
Male	430 (37.8)	58.7	30.6	
Residency				
Australian citizen/resident	969 (85.6)	85.5	84.1	.753
Other	163 (14.4)	14.5	15.9	
International student				
Yes	158 (14.1)	14.0	15.9	.917
No	964 (85.9)	86.0	84.1	
Study level				
Undergraduate	984 (87.5)	87.7	82.9	.480
Graduate/ Post-graduate	140 (12.4)	12.3	17.1	
Other	1 (0.1)	0.0	0.0	
Highest level of parents education				
High school or below	253 (22.4)	21.7	25.2	.588
Post-secondary school	869 (76.8)	77.4	74.8	
Other	10 (0.8)	0.9	0.0	
Hours of paid work during semester				
Zero hours	240 (21.1)	22.1	17.9	.865
1–25 h	636 (56.0)	55.2	56.5	
> 25 h	70 (6.2)	6.1	7.9	
Hours vary weekly	73 (6.4)	6.5	5.3	
Do not work during semester	117 (10.3)	10.1	12.4	
Years studying at university				
Less than 1 year	232 (20.6)	20.9	14.3	.207
1–3 years	455 (40.5)	40.6	34.5	
3–5 years	329 (29.3)	29.2	37.8	
More than 5 years	108 (9.6)	9.3	13.4	
Current study load				
Full-time	1033 (92.0)	92.0	91.9	.943
Part-time	81 (7.2)	7.2	7.0	
Withdrawn/ Deferred	9 (0.8)	0.8	1.1	
Mode of study				
On-campus	1054 (94.4)	94.6	93.0	.586
Off-campus/online student	63 (5.6)	5.4	7.0	
Living on campus				
Yes	102 (10.7)	10.6	9.8	.858
No	849 (89.3)	89.4	90.2	
Current Grade Point Average (GPA)				
Fail	16 (1.4)	1.4	4.3	.215
Pass (4.0–4.9)	168 (15.0)	15.2	18.7	
Credit (5.0–5.9)	416 (37.0)	37.2	31.1	
Distinction (6.0–6.9)	376 (33.4)	32.7	38.9	
High Distinction (7.0)	92 (8.2)	8.4	4.0	
Other	56 (5.0)	5.1	3.0	
Physical health problem diagnosis				
Yes	198 (17.4)	16.9	17.8	.428
No	938 (82.6)	83.1	82.2	
Mental health problem diagnosis				
Yes	227 (20.4)	19.0	32.1	.064
No	887 (79.6)	81.0	67.9	
Prescription for pharmaceutical stimulants				
Yes	16 (1.4)	0.8	11.8	< .001
No	1094 (98.6)	99.2	88.2	
Previous illicit drug use				
Yes	286 (25.9)	22.9	77.5	< .001
No	818 (74.1)	77.1	22.5	
Frequency of alcohol use				
Never/ Less than yearly	237 (21.5)	22.4	7.7	< .001
Yearly	228 (20.6)	20.9	10.9	
Monthly	365 (33.0)	32.7	39.0	
Weekly or more frequent	275 (24.9)	24.0	42.4	
Peer use of prescription stimulants to improve academic performance				
Yes	369 (34.8)	32.1	81.8	< .001
No	691 (65.2)	67.9	18.2	

*Weighted analyses were performed to examine the association between life time prescription stimulant use and sample characteristics. *P*-values were calculated based on designed based F-statistics

Participants had a mean age of 21.29 years (SD 2.78). Most had completed one year or more of their studies, were completing their studies full-time, and were completing an undergraduate degree. Small proportions were off-campus/online students, living on campus, or international students. Approximately a fifth of the sample indicated that they had been diagnosed with a physical health condition and a fifth of the sample had a psychological health condition. Sixteen students indicated that they had prescriptions for stimulant medications. One-quarter (25.9%; *n* = 286) of the sample reported that they had used an illicit drug for recreational purposes at some point.

### Prevalence of prescription stimulant use

Overall, lifetime use of any prescription stimulant was 6.5% (*n* = 74), with both past year use at 4.4% (*n* = 50) and repeat use (more than ‘once or twice only’) at 4.7% (*n* = 53). Students reported the use of a number of prescription stimulants to improve academic performance, which are displayed across stimulant types and time in Table 2.

**Table 2:** Weighted prevalence of prescription stimulant use to improve academic performance

Prescription stimulant	LifetimeUse n (%)	Past Year Use n (%)	Past 6 months n (%)	Past 3 months n (%)	Past Month n (%)	Past Week n (%)
Modafinil	30 (2.7%)	27 (2.3%)	23 (2.0%)	21 (1.8%)	17 (1.5%)	12 (1.0%)
Adderall	33 (2.9%)	19 (1.6%)	16 (1.4%)	13 (1.2%)	11 (1.0%)	5 (0.4%)
Concerta/Ritalin	29 (2.6%)	16 (1.5%)	12 (1.1%)	11 (1.0%)	10 (0.9%)	9 (0.8%)
Racetams	14 (1.2%)	9 (0.8%)	7 (0.6%)	7 (0.6%)	6 (0.5%)	3 (0.3%)
Atomoxetine	1 (0.1%)	0 (0.0%)	0 (0.0%)	0 (0.0%)	0 (0.0%)	0 (0.0%)
Phentermine	1 (0.1%)	1 (0.1%)	1 (0.1%)	1 (0.1%)	1 (0.01%)	1 (0.1%)

### Other substance use to improve academic performance

Legally available substances that were also reportedly used to improve academic performance had a lifetime prevalence of 46.6% (*n* = 529). The ten most commonly used legally available substances were: coffee (41.4%, *n* = 470), tea (25.9%, *n* = 294), energy drinks (23.6%, *n* = 268), cola (15.8%, *n* = 179), caffeine pills (9.1%, *n* = 104), Omega 3 supplements (7.4%, *n* = 84), alcohol (6.8%, *n* = 78), tobacco (4.3%, *n =* 49), cold and flu tablets (4.0%, *n* = 46), and Gingko biloba (3.1%, *n* = 35). The lifetime use of illicit substances to improve academic performance was 2.8% (*n* = 32) with the most commonly used substances being: cannabis (1.5%, *n* = 17), ice and speed (0.6%, *n* = 7) or LSD (0.5%, *n* = 6).

### Correlates of prescription stimulant use to improve academic performance

Binary logistic regression was used to examine the association between lifetime prescription stimulant use to improve academic performance and factors such as age, sex, and whether the participant had a prescription for stimulants, associated with other prescription stimulant users, frequency of alcohol use and history of illicit drug use. The factors that were associated with use of prescription stimulants to improve academic performance were: being male, OR = 2.46, 95% CI [1.38, 4.43], having a prescription for stimulants, OR = 33.66, 95% CI [6.28, 180.43], associating with other prescription stimulant users, OR = 4.25, 95% CI [2.19, 8.25], and previous illicit recreational drug use, OR = 7.99, 95% CI [3.80, 16.79]. Age and frequency of alcohol use were not significantly associated with the use of prescription stimulants to improve academic performance.

## Discussion

Contrary to reports that use of ‘study drugs’ is high [23], this study showed that both lifetime and recent use of prescription stimulants to improve academic performance was not common among Australian university students. Use was unsurprisingly higher among those with a prescription for stimulants, among those who associated with others who used prescription stimulants to improve their academic performance, and among male students.

These findings are important because they confirm the importance of access to prescription stimulants for use, whether this is through the ability to arrange a prescription for oneself, or via social networks. This is in keeping with other international studies [15, 17]. Another important finding was that non-medical use of prescription stimulants was strongly associated with a history of illicit drug use, and this is also consistent with previous international research [13, 15]. Neither age or alcohol use was associated with non-medical use of prescription stimulants to improve academic performance and these findings differ from previous US [20] and UK [17] studies that have shown alcohol use to be important, perhaps because the legal drinking age is 21 years in the US. This underscores the importance of country-specific studies. Another difference between US and Australia is the absence of the Greek system of sorority and fraternity houses in Australia. Membership of a sorority or fraternity house has been found to be associated with higher rates of non-medical use of prescription stimulants for academic purposes, particularly among males [9, 17]. The present study found that males were more likely to be users of prescription stimulants, but those living on campus were no more likely to do so than those living off campus. Self-reported Grade Point Average and health problems were also not associated with prescription stimulant use. These findings are consistent with previous longitudinal research showing no significant change over time in Grade Point Average among students who used prescription stimulants for nonmedical purposes but a significant increase among students who did not [27]. These results support the recommendation that prevention and intervention strategies should emphasise the lack of evidence for claims of academic benefits from non-medical use of prescription stimulants.

Prevalence estimates have varied widely between countries. In the current study, the prevalence of non-medical prescription stimulants use for study purposes was lower than other Australian [3] and international studies [10, 11]. However, in accordance with previous research, non-medical prescription stimulant use was a small fraction of the use of other legally available substances such as caffeine and energy drinks. This suggests that the focus on prescription stimulant use may be diverting attention from other potentially risky substance use by students to improve their academic performance. It also highlights the importance of not exaggerating the use of prescription stimulants for academic improvement as a common practice [28, 29].

There are a number of limitations that should be considered in interpreting the results of this study. The cross-sectional design means that it is not possible to infer causal relationships between the use of prescription stimulants and other factors. The use of self-report measures may have introduced recall and social desirability biases. The recruitment method may have introduced selection bias whereby students who were using prescription stimulants or illicit drugs were less likely to participate. Another limitation of the study is the non-representative sample due to the reliance on a convenience sample of students. Universities are increasingly regulating student surveys online, given the many surveys students are already asked to complete (e.g., evaluations of teaching) so population surveys of university students are difficult to conduct. The study also relied on participants to make the distinction between use of prescription stimulants for study purposes and use for recreational or other purposes but the survey explicitly reminded participants of the distinction. Surveys in other student populations have been subject to similar limitations and biases. It is also important to note that our sample included both undergraduate and postgraduate students within the age range. There were too few postgraduates to analyse differences between undergraduates and postgraduates in prescription stimulant use but other studies have found that older students and postgraduates are more likely to be users of prescription stimulants [17]. This would be a useful avenue of inquiry for further research in Australia.

## Conclusion

Further studies are needed to confirm the low prevalence of prescription stimulant use to improve academic performance among Australian students and to confirm the factors associated with such use. Until further evidence suggests otherwise there does not appear to be a strong case for specific policies or interventions targeting risky prescription stimulant use for improving academic performance among Australian students.

## Abbreviation

(ADHD): Attention Deficit Hyperactivity Disorder
